# Oncogenic EGFR mutants differentially alter dimerization, adaptor protein engagement, and clathrin-mediated internalization

**DOI:** 10.1016/j.jbc.2026.113353

**Published:** 2026-07-21

**Authors:** Maryam Bello-Hassan, Pradeep Kumar Singh, Adam W. Smith

**Affiliations:** Department of Chemistry & Biochemistry, Texas Tech University, Lubbock, Texas, USA

**Keywords:** PIE-FCCS, plasma membrane organization, clathrin adaptor recruitment, receptor multimerization, mutation-specific trafficking, live-cell fluorescence fluctuation spectroscopy, AP-2 dynamics, ligand-induced receptor clustering, membrane receptor organization, oncogenic signaling, endocytic routing, total internal reflection fluorescence microscopy, receptor internalization kinetics, non-small cell lung cancer mutations

## Abstract

The epidermal growth factor receptor (EGFR) is a receptor tyrosine kinase whose activation, clustering, and endocytic trafficking together regulate the duration and strength of downstream signaling. In non-small cell lung cancer (NSCLC), activating mutations such as exon 19 deletions (ex19del) and L858R drive oncogenesis, yet how these mutations alter receptor clustering and engagement with the endocytic machinery at the plasma membrane remains poorly understood. Here, we combined pulsed interleaved excitation fluorescence cross-correlation spectroscopy (PIE-FCCS) with quantitative live-cell total internal reflection fluorescence microscopy (TIRFM) to determine how EGFR variants differ in dimerization, adaptor recruitment, and internalization. PIE-FCCS measurements revealed mutation-specific differences in receptor clustering: EGFR^WT^ displayed ligand-induced multimerization, EGFR^L858R^ exhibited strong ligand-independent dimerization with additional ligand-driven clustering, whereas the exon 19 deletion variants showed weaker ligand-dependent dimerization and limited higher-order multimerization. Live-cell imaging of EGFR and the clathrin adaptor AP-2 during the first 30 min after EGF stimulation revealed distinct alterations in clathrin-mediated endocytosis (CME). The EGFR^E746–A750del^ mutant showed minimal AP-2 engagement, indicating impaired adaptor recruitment, whereas EGFR^L747–A750>P^ exhibited high basal AP-2 engagement that persisted after stimulation, suggesting altered clathrin assembly dynamics with sustained membrane association. In contrast, EGFR^L858R^ showed ligand-dependent AP-2 recruitment similar to wild type but with reduced net internalization. Together, these results demonstrate that oncogenic EGFR mutations produce distinct combinations of receptor clustering and adaptor engagement that differentially perturb early steps of CME. These findings provide mechanistic insight into how altered receptor dynamics at the plasma membrane may contribute to sustained signaling in RTK-driven cancers.

The epidermal growth factor receptor (EGFR) is a receptor tyrosine kinase that regulates cell proliferation, differentiation, and survival. Dysregulated EGFR signaling is a key driver of non-small cell lung cancer (NSCLC), the most common lung cancer subtype (1, 2). The most commonly occurring EGFR mutations in NSCLC include exon 19 deletions (*e*.*g*., E746–A750del, L747–A750 > P) and the L858R substitution, which constitutively activate kinase function and drive tumorigenesis through sustained PI3K/AKT and STAT signaling (3). Patients harboring these mutations are commonly treated with tyrosine kinase inhibitors (TKIs); however, clinical responses vary widely, and resistance to treatment frequently develops (4). These challenges highlight a critical need to better understand the mechanistic regulation of EGFR signaling.

Endocytosis is a key regulator of EGFR signaling because it controls receptor availability at the plasma membrane and influences receptor fate following activation. In wild-type (WT) EGFR, ligand binding triggers clathrin-mediated endocytosis (CME), a process initiated by recruitment of adaptor protein AP-2 and clathrin. This is accompanied by receptor ubiquitylation, which serves as a critical signal for subsequent endosomal sorting and eventual lysosomal degradation (5). Clathrin-coated structures have also been shown to function as spatially organized signaling platforms where receptor clustering and signaling can occur concurrently with endocytic engagement (6). Although CME is considered the predominant pathway for EGFR internalization under basal or moderate ligand stimulation, EGFR can also be internalized through clathrin-independent mechanisms. These alternative routes are typically engaged under conditions of elevated ligand concentrations, or cellular stress, and often display different kinetics and trafficking outcomes relative to CME (7, 8, 9, 10). In contrast to WT receptors, oncogenic EGFR mutants frequently display aberrant trafficking behavior, including impaired ubiquitylation, defective lysosomal targeting, and increased recycling, leading to prolonged signaling and enhanced oncogenic potential (11, 12, 13). However, most existing studies have focused on bulk internalization or downstream trafficking events, leaving the earliest steps of internalization, particularly adaptor protein recruitment and CME initiation, poorly understood.

Here, we use quantitative live-cell imaging with total internal reflection fluorescence microscopy (TIRFM) to investigate the early internalization dynamics of WT EGFR and clinically relevant mutants. By combining surface fluorescence intensity measurements with dual-color colocalization analysis of EGFR and AP-2, we directly examine how different EGFR mutations affect adaptor recruitment and internalization at the plasma membrane. We further compare these dynamics with receptor clustering measurements obtained by pulsed interleaved excitation fluorescence cross-correlation spectroscopy (PIE-FCCS), extending previous work from our group to include additional mutants. Together, these approaches reveal mutation-specific differences in EGFR clustering at the plasma membrane, AP-2 recruitment, and internalization dynamics, providing mechanistic insight into how oncogenic EGFR mutations alter receptor trafficking and may contribute to sustained signaling in NSCLC.

## Results

### Oncogenic EGFR mutants show different levels of dimerization and multimerization

**Figure 1:**
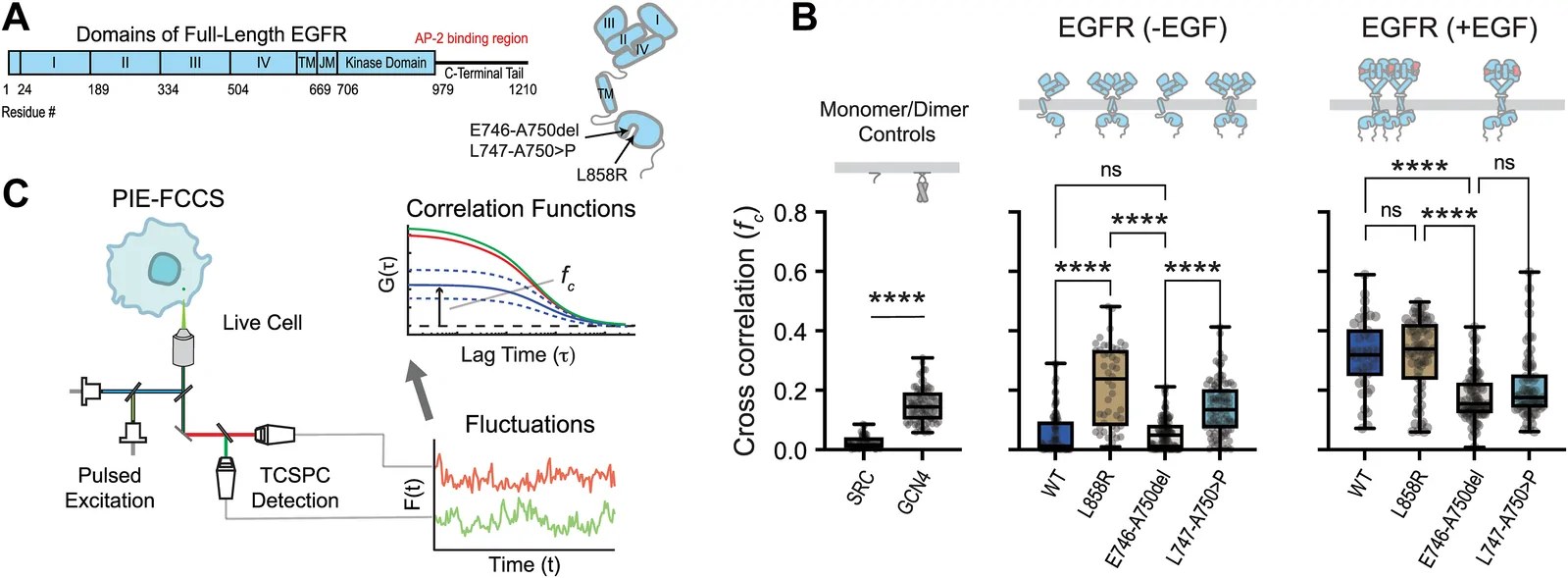
**Live-cell PIE-FCCS analysis of EGFR dimerization and multimerization.***A*, schematic representation of EGFR domain structure: The full-length EGFR protein is depicted, comprising the extracellular domains (I–IV), transmembrane, juxtamembrane, kinase, and C-terminal tail domains. The Y974RAL AP-2 binding motif, located within the C-terminal tail, is highlighted. A cartoon illustration next to the domain map depicts the folded arrangement of the extracellular domains (I–IV) relative to the kinase domain. Key activating mutations (E746-A750del, L747-A750 > P, and L858R) are located within the kinase domain. *B*, schematic of the PIE-FCCS instrument and workflow. Pulsed lasers alternately excite a diffraction-limited region within a live cell, and time-correlated single-photon counting (TCSPC) is used to record fluorescence intensity traces. These fluctuations are processed into auto- and cross-correlation functions, from which the relative cross-correlation amplitude (*f*_*c*_) is calculated as a measure of receptor dimerization or multimerization. *C*, PIE-FCCS measurements for monomer and dimer controls (*left*) and for EGFR^WT^, and three oncogenic mutants (EGFR^L858R^, EGFR^E746–A750del^, EGFR^L747–A750>P^) under basal (–EGF) and stimulated (+EGF) conditions. Box plots display the median with interquartile range; whiskers extend from minimum to maximum; individual data points are overlaid. Data consists of at least 40 cells from three independent experiments. Groups were compared by Kruskal-Wallis test followed by Dunn's multiple comparisons test. Under basal conditions, EGFR^L858R^ and EGFR^L747–A750>P^ exhibited significantly elevated *f*_*c*_ relative to EGFR^WT^ (*p* < 0.0001 for both), consistent with ligand-independent dimerization, whereas EGFR^E746–A750del^ was not significantly different from EGFR^WT^ (*p* > 0.9999). EGFR^L858R^ and EGFR^E746–A750del^ differed significantly from each other (*p* < 0.0001), as did EGFR^E746–A750del^ and EGFR^L747–A750>P^ (*p* < 0.0001), while EGFR^L858R^ and EGFR^L747–A750>P^ were not significantly different (*p* = 0.3626). Following EGF stimulation, EGFR^WT^ and EGFR^L858R^ showed comparable *f*_*c*_ values (*p* > 0.9999), whereas both exon 19 deletion mutants displayed significantly lower *f*_*c*_ than EGFR^WT^ (*p* < 0.0001 for EGFR^E746–A750del^; *p* = 0.0002 for EGFR^L747–A750>P^) and EGFR^L858R^ (*p* < 0.0001 for both), indicating limited ligand-induced multimerization. EGFR^E746–A750del^ and EGFR^L747–A750>P^ were not significantly different from each other after EGF stimulation (*p* = 0.7538).

To determine how activating EGFR mutations alter receptor–receptor interactions at the plasma membrane, we used live-cell PIE-FCCS to quantify receptor co-diffusion under basal conditions and following ligand stimulation (Fig. 1). In PIE-FCCS, fluorescence intensity fluctuations from independently excited green and red fluorophores are used to calculate auto-correlation and cross-correlation functions. The extent of receptor co-diffusion is quantified using the relative cross-correlation amplitude (*f*_*c*_), which reflects the fraction of receptors moving together within the observation area (Fig. 1*C*). Higher *f*_*c*_ values therefore indicate increased receptor dimerization or higher order multimerization at the plasma membrane. Under basal (–EGF) conditions, EGFR^WT^ displayed low *f*_*c*_ values, consistent with a predominantly monomeric population at the cell surface with only a small subset of pre-existing dimers (Fig. 1B) as reported previously (14). In contrast, the EGFR^L858R^ mutant exhibited substantially elevated *f*_*c*_ values, indicating robust ligand-independent dimerization (Fig. 1B). This is consistent with prior single molecule tracking studies that also reported elevated dimerization of EGFR^L858R^ (15). The EGFR^L747–A750>P^ mutant also demonstrated enhanced basal *f*_*c*_, albeit to a lesser extent than EGFR^L858R^, whereas the EGFR^E746–A750del^ variant showed *f*_*c*_ values only slightly above those of wild type. These results indicate that the two exon 19 deletion mutants differ markedly in their ability to form stable dimers or oligomers, despite arising from alterations within the same region of the kinase domain as reported previously (16). Spearman correlation analysis confirmed that *f*_*c*_ values were largely independent of receptor surface density for EGFR^WT^, EGFR^E746–A750del^, and EGFR^L747–A750>P^ (Fig. S1). A modest negative correlation was observed for EGFR^L858R^; however, the elevated basal *f*_*c*_ values of this mutant could not be explained solely by increased receptor density.

EGF stimulation produced distinct ligand-dependent responses across the variants. As expected, EGFR^WT^ showed a pronounced increase in *f*_*c*_ following ligand addition, reaching uniformly high values indicative of ligand-driven dimerization and multimerization (Fig. 1*B*) (14). EGFR^L858R^, which was already strongly dimeric at baseline, exhibited a modest increase in *f*_*c*_, but to a mean level nearly identical to EGFR^WT^ (Fig. 1*B*). These results suggest that EGFR^L858R^ was already partially multimeric prior to ligand stimulation. Both exon 19 deletion mutants demonstrated ligand-induced increases in *f*_*c*_; however, their maximal *f*_*c*_ values remained substantially lower than those of wild type or EGFR^L858R^. EGFR^E746–A750del^ showed the weakest ligand response, whereas EGFR^L747–A750>P^ achieved intermediate *f*_*c*_ values consistent with partial multimerization as reported previously (16).

Together, these PIE-FCCS measurements reveal that oncogenic EGFR mutations produce distinct dimerization signatures at the plasma membrane, ranging from strongly constitutive (EGFR^L858R^), to partially constitutive (EGFR^L747–A750>P^), and to weak and ligand-limited (EGFR^E746–A750del^). As the extent and stability of EGFR dimers influence not only kinase activation but also the recruitment of endocytic adaptor proteins, these differences in ligand-induced multimerization suggest that there could be mutation-specific effects on downstream trafficking pathways described below.

### EGFR mutants display impaired internalization

While previous studies have established that NSCLC-associated EGFR mutants undergo constitutive internalization and aberrant intracellular sorting (11), the initial association of these receptors with the endocytic machinery at the cell surface remains less clearly defined. Here, we utilized TIRFM to selectively visualize the plasma membrane interface, enabling a comparative measure of receptor recruitment to the AP-2 complex at the plasma membrane.

**Figure 2:**
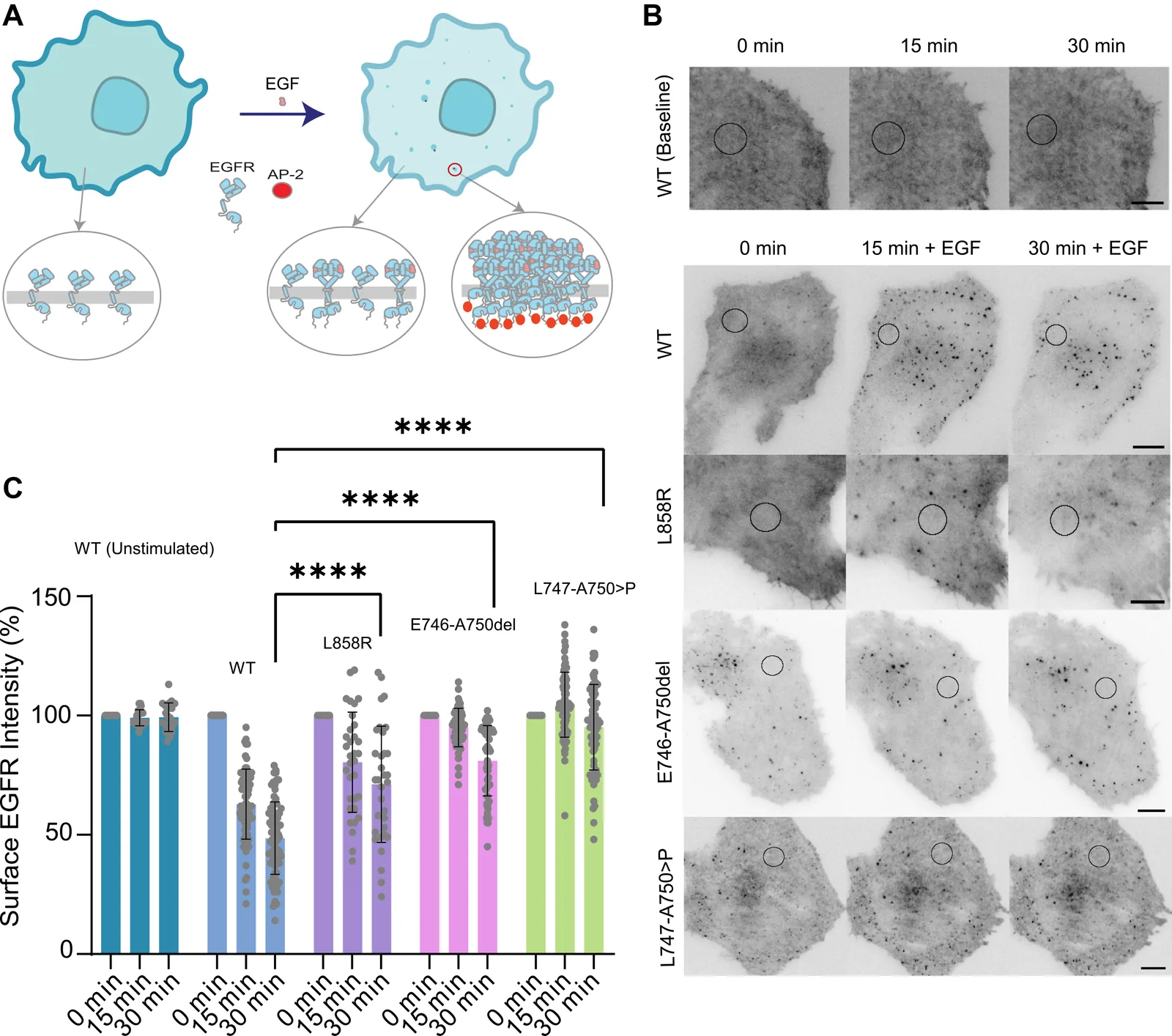
**EGFR displays a mutant-sensitive reduction in surface intensity after EGF stimulation.***A*, schematic of the TIRFM-based quantification workflow. The *left* cell shows EGFR predominantly as freely diffusing monomers at the plasma membrane, devoid of puncta. Following EGF stimulation, the right cell shows discrete puncta colocalizing with AP-2, consistent with endocytic clusters containing activated EGFR, accompanied by a corresponding reduction in freely diffusing monomers. ROIs were drawn on puncta-free membrane regions for mean intensity measurement; background fluorescence from cell-free regions was subtracted prior to quantification. *B*, representative TIRFM images of SUM159 cells expressing EGFR^WT^ or mutant constructs, acquired 24 h post-transfection at 0, 15, and 30 min following stimulation with 100 ng/ml EGF. *C*, quantification of mean EGFR fluorescence intensity at the plasma membrane. Data points represent mean ± SD (n > 80 cells per construct from more than three independent experiments). Groups were compared by two-way repeated-measures ANOVA, followed by Dunnett's multiple comparisons test against WT-stimulated cells at each time point. At 30 min post-stimulation, all oncogenic mutants showed significantly reduced internalization compared to WT-stimulated cells (*p* < 0.0001 for E746–A750del, L747–A750 > P, and L858R). Similar significance was observed at 15 min (*p* < 0.0001 for E746–A750del and L747–A750 > P; *p* = 0.0003 for L858R).

To first validate our workflow, SUM159 cells expressing EGFR^WT^–eGFP were imaged live at baseline and after stimulation with 100 ng/ml EGF under TIRF illumination. Fluorescence intensities were measured per cell by averaging values from multiple ROIs at baseline and after 15 and 30 min of EGF stimulation, following background subtraction and normalization (See Experimental procedure). Imaging was performed on three independent experimental days, and single-cell membrane-associated fluorescence was quantified (n > 80 cells from three independent experiments). Representative images (Fig. 2*B*) and quantification (Fig. 2*C*) revealed a time-dependent decrease in EGFR intensity in cells expressing EGFR^WT^, with ∼40% and ∼55% reductions at 15 and 30 min, respectively, relative to baseline. We next compared this with three clinically relevant mutants: EGFR^L858R^ and two ex19del mutants (E746–A750del and L747–A750 > P). Baseline surface fluorescence intensities were not significantly different across constructs, indicating comparable receptor expression prior to ligand stimulation (Fig. S2). Spearman correlation analysis further confirmed that baseline EGFR expression levels did not account for the observed differences in receptor downregulation across variants, although a weak correlation was noted for EGFR^L858R^ (Fig. S3). EGFR^L858R^ exhibited a smaller but significant decrease (∼30% and ∼40% at 15 and 30 min), whereas EGFR^E746–A750del^ showed only a modest ∼20% reduction at 30 min and no significant change at 15 min. Strikingly, EGFR^L747–A750>P^ showed no significant reduction at either time point, indicating a near-complete defect in ligand-induced internalization. Control experiments in unstimulated cells confirmed that EGFR fluorescence intensity remained stable throughout the imaging period (Fig. S4). Statistical analysis by two-way ANOVA with *post hoc* multiple comparisons confirmed that the reduction in WT cells was significant and greater than that observed for all mutants at both 15 and 30 min. Together, these results demonstrate that EGFR mutants display impaired internalization compared with WT, with the degree of impairment varying by mutation.

### Ligand-dependent association of EGFR^WT^ with CME adaptor protein (AP-2)

**Figure 3:**
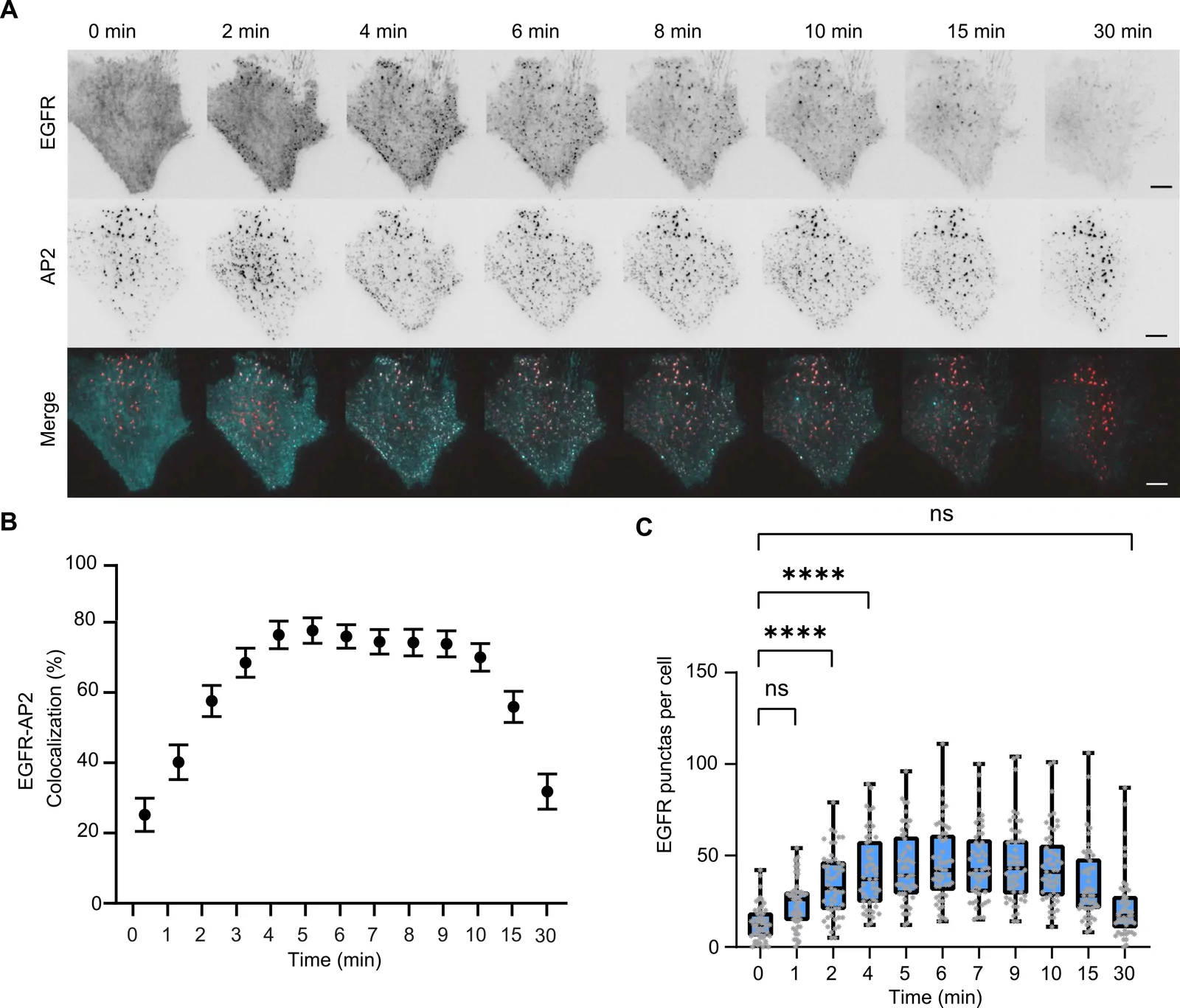
**EGFR displays time dependent AP-2 interaction following EGF stimulation.***A*, representative dual-color TIRFM images of SUM159 cells transiently expressing EGFR–eGFP (*cyan*) and endogenously expressing AP-2–HaloTag (*red*) at baseline (0 min) and the indicated times after stimulation with 100 ng/ml EGF. Scale bar, 10 μm. *B*, quantification of EGFR–AP-2 colocalization, expressed as the percentage of EGFR puncta with an AP-2 punctum (≤0.4 μm). *C*, quantification of EGFR puncta per cell at the indicated time points. Box plots display the mean with interquartile range; whiskers extend from minimum to maximum. Data represents > 50 cells from three independent experiments. Statistical significance was assessed using a one-way ANOVA followed by Tukey's multiple comparisons test against the 0 min time point; *p* < 0.0001 at 2 and 4 min, while 1 min (*p* = 0.0733) and 30 min (*p* = 0.3127) did not differ significantly from 0 min.

Following ligand stimulation, EGFR is internalized primarily through CME, a process requiring the adaptor AP-2 to recruit clathrin and other accessory proteins (17, 18, 19, 20). To monitor CME dynamics in real time, we performed dual-color TIRFM in SUM159 cells endogenously expressing AP-2 fused to a HaloTag (21), and transiently transfected with EGFR^WT^–eGFP. This enabled simultaneous visualization of EGFR and AP-2 puncta at the plasma membrane. Colocalization was quantified using a nearest-neighbor algorithm, where EGFR puncta were classified as colocalized if an AP-2 punctum was detected within the defined distance threshold (see Experimental procedure). Colocalization percentage was calculated as the fraction of colocalized EGFR puncta relative to total EGFR puncta per cell at each time point. Cells were imaged at baseline, every minute for the first 10 min, and subsequently at 15 and 30 min following 100 ng/ml EGF stimulation. Representative images (Fig. 3*A*) show minimal EGFR puncta at baseline, followed by a rapid formation and recruitment of EGFR puncta to AP-2-positive structures within the first 2 min of stimulation. Quantification across >50 cells from three independent experiments revealed a robust increase in puncta number, peaking at ∼5 to 6 min and declining progressively thereafter (Fig. 3*C*). Colocalization with AP-2 followed a similar trajectory, reaching its maximum during the early phase and decreasing toward baseline levels by 30 min (Fig. 3*B*). Statistical analysis by one-way ANOVA with *post hoc* multiple comparisons confirmed that both puncta numbers and colocalization percentages were significantly elevated relative to baseline during the first 2 to 10 min (∗∗∗*p* < 0.001) but were no longer statistically different at 30 min. Together, these results confirm that EGFR^WT^ undergoes efficient CME-dependent internalization following ligand stimulation and validate the critical role of AP-2 in mediating this process.

### EGFR mutants exhibit distinct CME adaptor recruitment dynamics

To determine whether impaired internalization of EGFR mutants reflects altered adaptor protein recruitment and CME initiation, we quantified EGFR–AP-2 colocalization dynamics for three mutants (L858R, E746–A750del, and L747–A750 > P) using dual-color TIRFM and compared the results with those of EGFR^WT^ characterized above.

**Figure 4:**
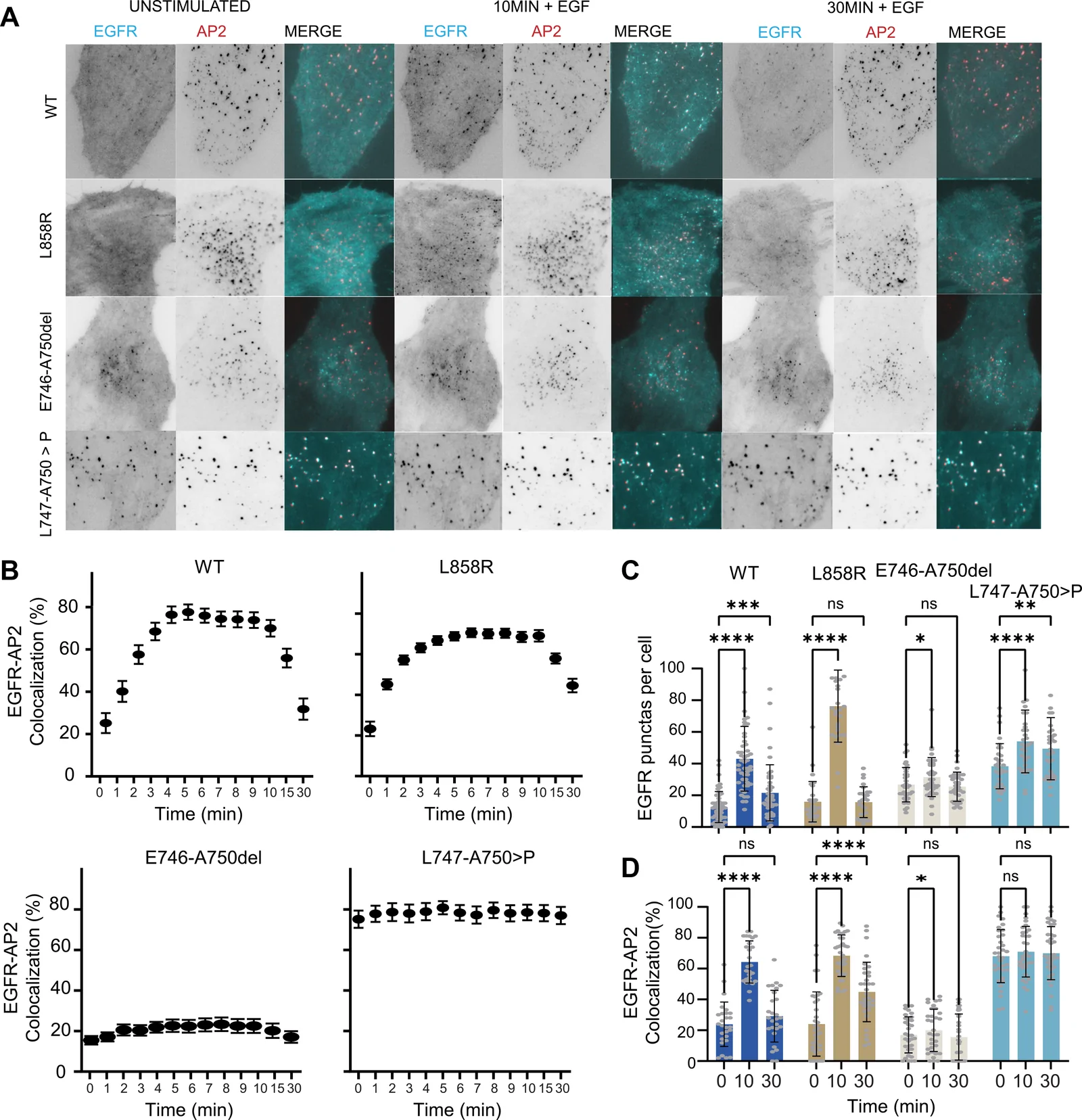
**EGFR mutants display distinct AP-2 recruitment and puncta dynamics.***A*, representative dual-color TIRFM images of SUM159 cells expressing EGFR–eGFP constructs (WT, L858R, E746–A750del, L747–A750 > P) together with endogenously tagged AP-2 at baseline (0 min), 10 min and 30 min after stimulation with 100 ng/ml EGF. Scale bar, 10 μm. *B*, full time-course profile of EGFR–AP-2 colocalization from 0 to 10 min (1-min intervals) with additional measurements at 15 and 30 min. Data represents more than 40 cells per construct from three independent experiments. *C*, quantification of total EGFR puncta per cell at 0-, 10-, and 30-min post-stimulation. Data represent mean ± SD (n > 40 cells per condition from three independent experiments). Statistical significance was assessed by two-way ANOVA followed by Dunnett's multiple comparisons test against 0 min within each construct. WT showed significant changes at both 10 min (*p* < 0.0001) and 30 min (*p* = 0.0002); L858R showed a significant increase at 10 min (*p* < 0.0001); E746–A750del showed a significant increase at 10 min (*p* = 0.0154); L747–A750 > P showed significant changes at both 10 min (*p* < 0.0001) and 30 min (*p* = 0.0010). *D*, quantification of EGFR–AP-2 colocalization at 0-, 10-, and 30-min post-stimulation. Statistical significance was assessed by two-way ANOVA followed by Dunnett's multiple comparisons test against 0 min within each construct. WT and L858R showed significant increases at 10 min (*p* < 0.0001 for both), while E746–A750del showed a modest but significant increase (*p* = 0.0230); L747–A750 > P showed no significant change at either time point.

Representative images (Fig. 4*A*) show various EGFR constructs and AP-2 at baseline (0 min), 10 min, and 30 min after EGF stimulation, with the full time-lapse series (0–10 min at 1-min intervals, plus 15 and 30 min) provided in Figures S5–S8. Quantification across at least 40 cells per construct from three independent experiments (Fig. 4, *B*–*D*) revealed distinct patterns for each mutant. EGFR^L858R^ behaved similarly to EGFR^WT^, showing a strong increase in puncta number and colocalization after EGF stimulation. However, in contrast to EGFR^WT^, EGFR^L858R^ maintained significantly elevated colocalization at 30 min even as puncta counts returned to baseline, suggesting prolonged residence of a subset of EGFR puncta in AP-2–positive structures.

Both EGFR ex19del variants displayed significantly more puncta at baseline than EGFR^WT^ (Fig. S9). For EGFR^E746–A750del^, Baseline puncta exhibited minimal AP-2 colocalization, and although EGF stimulation produced a slight increase in AP-2 association (from ∼15% at baseline to ∼23% at 10 min) along with a modest rise in puncta number, AP-2 recruitment remained markedly low throughout the 30-min time course, suggesting altered initiation of CME. In contrast, EGFR^L747–A750>P^ displayed numerous AP-2–colocalized puncta (∼67%) at baseline and exhibited a significant increase in puncta number after EGF stimulation. However, colocalization percentages remained high and largely unchanged throughout the 30-min imaging period. Statistical analysis confirmed these trends, revealing significant time-dependent responses for EGFR^WT^ and EGFR^L858R^, but not for either ex19del variant. Together, these results demonstrate that EGFR mutants differ not only in clustering and internalization efficiency but also in their engagement with CME machinery. While EGFR^L858R^ behaves similarly to EGFR^WT^, EGFR^E746–A750del^ displays impaired adaptor recruitment, and EGFR^L747–A750>P^ shows evidence of AP-2 engagement but appears to show limited progression through an early step in the CME pathway, resulting in sustained AP-2 colocalization even after prolonged stimulation.

## Discussion

In this study, we compared receptor clustering, internalization dynamics, and engagement with CME machinery for three clinically relevant oncogenic EGFR mutants (L858R, E746–A750del, and L747–A750 > P) relative to wild-type EGFR. All three mutants exhibited elevated basal phosphorylation relative to EGFR^WT^ in the absence of ligand stimulation (Fig. S10) consistent with previous reports (11). However, these EGFR mutations do not produce a single uniform defect in receptor clustering, adaptor engagement, and clathrin-mediated internalization. Using PIE-FCCS, we first quantified the dimerization and multimerization potential of each variant at the plasma membrane before and after ligand stimulation. We then combined TIRFM measurements of membrane fluorescence with dual-color imaging of the adaptor protein AP-2 to determine how these differences in receptor clustering relate to early events in CME-dependent internalization. Together, these complementary measurements allowed us to directly compare receptor dimerization state, ligand-induced internalization, and adaptor recruitment across EGFR variants under identical experimental conditions.

EGFR^WT^ undergoes rapid internalization *via* CME following ligand binding at the plasma membrane (18), a behavior we confirmed under our imaging conditions. In contrast, EGFR mutants exhibited heterogeneous internalization dynamics. The EGFR^L747–A750>P^ mutant showed no measurable decrease in fluorescence intensity at 30 min, whereas the canonical EGFR^E746–A750^ deletion displayed only a modest reduction. Notably, the EGFR^L858R^ mutant exhibited a clear decrease in surface intensity following EGF stimulation that was greater than the exon 19 mutants but did not reach the extent observed for wild type. This finding was initially surprising but is consistent with prior reports that these mutants differ in their multimerization behavior, which may influence receptor clustering and trafficking (16).

At baseline, both ex19del mutants exhibited elevated numbers of fluorescent puncta, consistent with previous studies showing that mutant EGFR localizes not only to the plasma membrane but also to endosomal compartments and undergoes constitutive internalization (11, 12). Our colocalization analysis revealed that EGFR^E746–A750del^ puncta were largely AP-2–negative and showed only minimal increases after EGF stimulation, indicating an impaired ligand induced CME initiation. By contrast, EGFR^L747–A750>P^ showed high AP-2 colocalization at baseline with only a slight increase after EGF stimulation, followed by sustained AP-2 colocalization throughout the time course. In comparison, EGFR^L858R^ demonstrated ligand-dependent AP-2 recruitment dynamics similar to the transient behavior observed for wild type, although with reduced overall surface loss. These findings suggest that while EGFR^E746–A750del^ shows impaired engagement with the clathrin adaptor machinery, EGFR^L747–A750>P^ remains persistently associated with it, and EGFR^L858R^ retains regulated adaptor engagement. That both exon 19 deletion mutants appear largely unresponsive to EGF in this context suggests a shared decoupling of ligand sensing from endocytic machinery recruitment, despite their otherwise distinct behaviors.

Endocytic adaptor proteins such as AP-2 recognize cargo through short tyrosine-based and dileucine-based sorting motifs within the cytoplasmic tails of receptors and engage clathrin through multivalent low-affinity interactions that depend strongly on how these motifs are spatially presented at the membrane rather than on a single high-affinity binding site (22). Because exon 19 deletions reside in the β3–αC loop of the kinase domain, a region shown to influence the positioning of intracellular elements of EGFR (23), these mutations are well positioned to alter how EGFR’s cytoplasmic sorting motifs are displayed to adaptor proteins without directly mutating the motifs themselves.

In this context, the behavior of the two mutants becomes more interpretable. The largely AP-2–negative puncta observed for EGFR^E746–A750del^ are consistent with impaired adaptor recognition, where sorting motifs may be insufficiently exposed for efficient AP-2 engagement. Conversely, the insertion of a proline in EGFR^L747–A750>P^ may introduce conformational constraints that promote persistent exposure of adaptor-recognition motifs, leading to constitutive AP-2 association. This may explain why EGFR^L747–A750>P^ engages CME machinery but appears unable to progress normally through endocytic dynamics, resulting in prolonged plasma membrane residency of EGFR–AP-2 assemblies.

**Figure 5:**
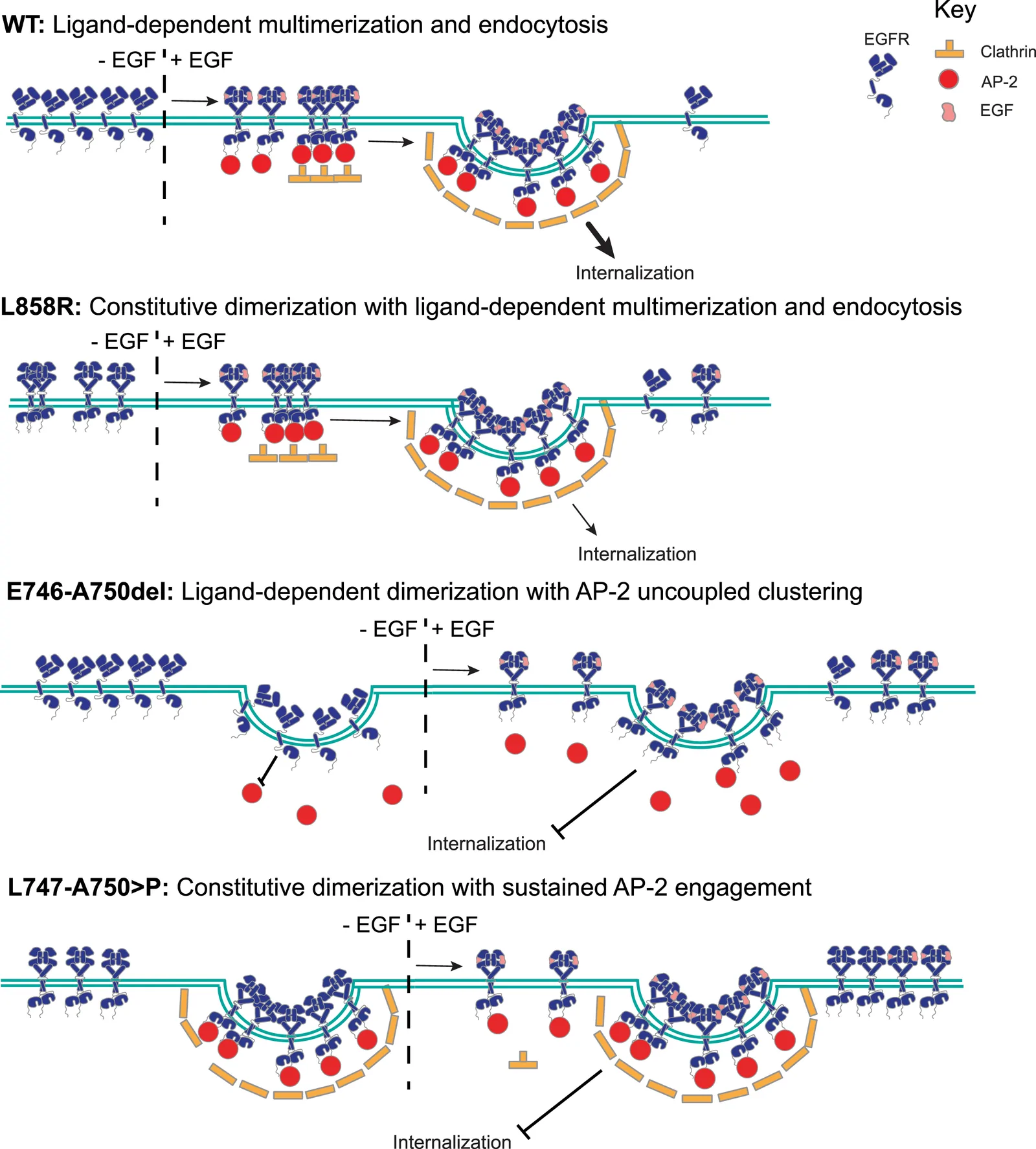
Model illustrating mutation-specific differences in EGFR activation, clustering, AP-2 recruitment, and internalization.

To integrate these observations, we propose a mechanistic model describing how distinct EGFR mutations alter receptor activation, clustering, and engagement with AP-2 (Fig. 5). In this framework, EGFR^WT^ exists predominantly as monomers at the plasma membrane prior to ligand stimulation. EGF binding induces receptor dimerization and multimerization, leading to kinase activation, recruitment of the adaptor protein AP-2, and efficient internalization.

The EGFR^L858R^ mutant differs in that receptors are already multimerized and constitutively active in the absence of ligand, yet they remain responsive to ligand stimulation. Upon EGF addition, EGFR^L858R^ recruits AP-2 and undergoes internalization, although to a lesser extent than WT, consistent with its partially dysregulated trafficking behavior.

In contrast, the exon 19 mutants disrupt these steps in distinct ways. The EGFR^E746-A750del^ mutant remains predominantly monomeric prior to ligand stimulation but is constitutively activated and forms puncta at the plasma membrane consistent with ongoing internalization. However, these puncta show little AP-2 association at baseline and only modest increases after ligand stimulation. Even when ligand induces receptor dimerization, AP-2 recruitment remains inefficient and internalization proceeds poorly.

The EGFR^L747–A750>P^ mutant exhibits a different phenotype. Receptors are predominantly dimerized and constitutively active prior to ligand stimulation and form puncta consistent with constitutive activation. Unlike EGFR^E746–A750del^, these puncta display strong AP-2 association even in the absence of ligand. Following ligand stimulation, receptors remain dimerized and persistently associated with AP-2, suggesting that EGFR–AP-2 assemblies form but fail to progress normally through endocytic maturation, resulting in stalled internalization.

Together, these findings demonstrate that oncogenic EGFR mutations do not produce a single uniform defect in endocytosis but instead generate distinct receptor organizational and trafficking states at the plasma membrane. L858R exhibited strong constitutive dimerization with preserved ligand-dependent AP-2 recruitment but reduced internalization, whereas the exon 19 deletion variants displayed distinct combinations of altered clustering and adaptor engagement. E746–A750del showed limited AP-2 recruitment and weak ligand-induced multimerization, while L747–A750 > P exhibited elevated basal clustering and persistent association with AP-2-positive structures consistent with altered endocytic progression. These results suggest that mutation-specific differences in receptor organization influence how EGFR engages the clathrin-mediated endocytic machinery and may contribute to sustained signaling in EGFR-driven cancers.

Although the current work establishes mutation-specific differences in EGFR organization and endocytic behavior, EGFR assembly is governed by multiple extracellular, transmembrane, juxtamembrane, and kinase-domain interfaces. Future studies using interface-specific EGFR mutants will be required to determine how these individual interaction surfaces contribute to adaptor engagement, receptor clustering, and clathrin-mediated internalization.

More broadly, these findings support an emerging view that receptor organization and endocytic routing are tightly coupled. By integrating live-cell PIE-FCCS measurements of receptor assembly with dynamic imaging of adaptor recruitment and internalization, this study provides a framework for understanding how oncogenic EGFR mutations remodel plasma membrane signaling states beyond kinase activation alone.

## Experimental procedures

### PIE-FCCS cell culture and sample preparation

COS-7 cells were used for PIE-FCCS measurements of EGFR receptor interactions at the plasma membrane. Cells were cultured in Dulbecco’s Modified Eagle’s Medium (DMEM) supplemented with 10% fetal bovine serum and 1% penicillin–streptomycin. For imaging experiments, cells were seeded onto 35-mm glass-bottom MatTek dishes and transfected at approximately 70 to 80% confluence.

EGFR constructs were subcloned into eGFP-N1 and mCherry-N1 expression vectors using XhoI and AgeI restriction sites to generate C-terminally tagged fluorescent proteins. Cells were transiently transfected approximately 20 h before imaging using Lipofectamine 2000 (Thermo Fisher Scientific). For PIE-FCCS measurements, cells were co-transfected with equal amounts of eGFP- and mCherry-tagged receptor plasmids (1:1 ratio; total DNA 2.5 μg per dish) to ensure balanced expression of both fluorophore-labeled populations. Under these conditions, receptor densities at the plasma membrane ranged from approximately 100 to 1200 receptors μm^−2^.

Immediately prior to imaging, the culture medium was replaced with phenol-red–free Opti-MEM I reduced serum medium (Thermo Fisher Scientific). Measurements were performed either in the absence of ligand or following stimulation with recombinant human EGF. For ligand stimulation experiments, a stock solution of EGF (20 μg/ml) was diluted to 500 ng/ml in imaging medium and added to cells approximately 15 min prior to data collection. Measurements were collected for up to one hour following ligand addition.

### PIE-FCCS instrumentation

Fluorescence correlation spectroscopy measurements were performed using a custom-built inverted confocal microscope equipped for pulsed interleaved excitation and time-correlated single photon counting described previously. Excitation was provided by a supercontinuum pulsed laser (SuperK, NKT Photonics) operating at a repetition rate of 9.2 MHz. The beam was split into two excitation wavelengths (488 nm and 561 nm) using a series of filters and mirrors.

To generate pulsed interleaved excitation, the two beams were routed through optical fibers of different lengths to introduce a temporal delay between excitation pulses. The beams were recombined using a dichroic beam splitter and directed through a custom filter block before entering the microscope. The overlapped excitation beams were focused through a 100 × objective to produce a diffraction-limited observation volume at the peripheral plasma membrane of transfected cells.

Fluorescence emission was detected using avalanche photodiodes (Micro-Photon Devices) operating in time-tagged time-resolved mode. Photon arrival times were recorded using time-correlated single photon counting electronics. PIE gating of photon arrival times was used to eliminate spectral crosstalk between channels prior to correlation analysis.

### Calibration and control samples

System alignment and calibration were performed using fluorescently labeled DNA duplex standards to ensure proper overlap of the confocal volumes corresponding to the two excitation wavelengths. A 40-nucleotide duplex DNA labeled with TAMRA and FAM was used as a positive control for cross-correlation measurements. The labeled strand (TAMRA-40-FAM) was diluted to a final concentration of 100 nm in 10 mm TE buffer.

Membrane-anchored monomeric and dimeric peptide constructs were used as negative and positive controls for membrane measurements. The monomer control consisted of a lipidated Src peptide, whereas the dimer control contained the GCN4 leucine zipper motif fused to the same membrane anchor. These constructs established baseline *f*_*c*_ values corresponding to monomeric and dimeric states at the plasma membrane.

### Data acquisition

For cellular PIE-FCCS measurements, the excitation beams were positioned on flat peripheral regions of the plasma membrane to selectively measure membrane-associated receptors and avoid contributions from intracellular vesicles or organelles. Each measurement location was sampled six times with acquisition intervals of 10 s each.

Fluorescence intensity traces were recorded simultaneously for the green (eGFP) and red (mCherry) channels. Photon arrival times were gated based on the excitation pulse using PIE to assign photons to the appropriate excitation channel prior to correlation analysis.

### Correlation analysis

Auto-correlation and cross-correlation functions were calculated using custom MATLAB scripts. Intensity fluctuations were first divided into 10-s bins and then used to calculate time-dependent auto-correlation functions (ACFs) for each fluorophore channel and the cross-correlation function (CCF) between channels.

The autocorrelation function was defined as

$${G\left(\tau \right)=\left\langle F\left(t\right)\;F\left(t+\tau \right)\right\rangle /\;\left\langle F\left(t\right)\right\rangle }^{2}$$

and the cross-correlation function as

$$G_{GR}\left(\tau \right)=\left\langle F_{G}\left(t\right)\;F_{R}\left(t+\tau \right)\right\rangle \;/\;\left\langle F_{G}\left(t\right)\right\rangle \;\left\langle F_{R}\left(t\right)\right\rangle$$

The averaged autocorrelation curves from six acquisitions were fit to a two-dimensional diffusion model appropriate for membrane proteins that accounts for triplet relaxation and dark state dynamics:

$$G\left(\tau \right)=\left(1+\frac{T}{1-T}e^{-\tau /\tau_{T}}\right)\left(\frac{1}{\left\langle N\right\rangle }\right)\left(\frac{1}{1+\tau /\tau_{D}}\right)+1$$

where T represents the fraction of molecules in the triplet state, $\tau_{T}$ is the triplet relaxation time, ⟨N⟩ is the average number of molecules in the observation area, and $\tau_{D}$ is the characteristic diffusion time.

Effective diffusion coefficients were calculated using

$$D_{eff}=\frac{\omega^{2}}{4\tau_{D}}$$

where *ω* represents the lateral radius of the observation area.

The fraction of cross-correlation (*f*_*c*_) was calculated from the amplitudes of the auto- and cross-correlation functions and represents the fraction of receptors that co-diffuse within the observation area. Higher *f*_*c*_ values indicate increased receptor dimerization or multimerization at the plasma membrane.

### Cells and transfections

SUM159 cells were grown in 100-mm Petri dishes in a 1:1 mixture of high-glucose DMEM and Ham’s F-12 (Hyclone, GE Healthcare) supplemented with 5% fetal bovine serum (Hyclone), penicillin/streptomycin/L-glutamine (Hyclone), 1 μg/ml hydrocortisone (H4001; Sigma-Aldrich), 5 μg/ml insulin (I6634; Sigma-Aldrich), and 10 mM HEPES (Fisher Scientific), pH 7.4. Cultures were maintained at 37 °C in a humidified atmosphere with 5% CO_2_. At ∼90 to 100% confluency, cells were passaged and seeded onto fibronectin-coated 35-mm glass-bottom dishes (MatTek) in the same enriched medium. At ∼65 to 70% confluency, cells were transfected with EGFR-GFP constructs (wild-type, E746-A750del, L747-A750P, and L858R) using 4 μl of Lipofectamine 2000 (Invitrogen) diluted in 120 μl of phenol-free Opti-MEM (Thermo Fisher Scientific) according to the manufacturer’s recommendations. Experiments were performed 24 h post-transfection.

### Live-cell imaging

For the simultaneous visualization of EGFR and AP-2, SUM159 cells expressing EGFR-GFP and HaloTagged σ2 were first serum-starved in phenol-free Opti-MEM (Thermo Fisher Scientific) for 1 h at 37 °C. Following starvation, AP-2 was fluorescently labeled by incubating the cells with 50 nm Janelia Fluor 646-HaloTag ligand (Promega) for 10 min at 37 °C. Unbound ligand was removed by washing the cells with fresh prewarmed phenol-free Opti-MEM, and cells were subsequently maintained in the same medium for imaging. The cells were then either imaged immediately or subsequently after stimulation with 100 ng/ml EGF (Peprotech). Throughout all imaging and stimulation procedures, cells were maintained at 37 °C using an on-stage incubator integrated into the TIRFM system.

### TIRF microscopy

Time-lapse imaging was conducted on a fully motorized Nikon Eclipse Ti-E inverted microscope (Nikon Instruments) equipped with the Nikon Perfect Focus System and a TIRF illuminator. TIRF excitation was delivered *via* diode lasers (Coherent OBIS LX 488 nm, 100 mW; Coherent OBIS LS 561 nm, 40 mW; Coherent) through a 60 × 1.49 NA oil-immersion TIRF objective. Chromatic alignment was calibrated prior to each experiment using TetraSpeck beads (Thermo Fisher Scientific) to verify proper illumination and correct for aberrations (Fig. S11). Simultaneous two-color imaging of EGFR-eGFP and HaloTagged AP-2 utilized excitation at 488 nm and 561 nm, respectively. Emission signals were spectrally separated with a W-View Gemini image splitter and detected using a Flash 4.0 sCMOS camera (Hamamatsu Photonics). Total exposure time for each two-channel acquisition was 300 ms, with acquisition conditions kept constant across all quantitative experiments.

### Image processing and analysis

Fluorescence intensity measurements were analyzed using ImageJ software (National Institutes of Health, Bethesda, MD). Mean fluorescence intensity across the plasma membrane was determined by averaging multiple small circular regions of interest (ROIs) per cell. Background intensity was measured from cell-free regions and subtracted from corresponding cell intensities to yield the corrected intensity (I_corrected_ = I_ROI_-I_background_). Post-stimulation values were normalized to baseline and expressed as Surface EGFR intensity (%) using the formula: ((I_post_-I_baseline_)/I_baseline_).

Puncta detection and tracking were performed using the TrackMate plugin in Fiji (24). Tracking parameters were set to a maximum centroid displacement of 0.8 μm for frame-to-frame linking, with gap-closing permitted for puncta disappearing for two consecutive frames (2 min) if they reappeared within 0.8 μm of their previous position. Nearest-neighbor distances between EGFR (green) and AP-2 (red) puncta were calculated using a k-nearest neighbors (KNN) algorithm implemented in Python. Colocalization was defined as a center-to-center distance 0.4 μm, a threshold selected to account for diffraction-limited resolution, residual chromatic aberration, and localization error. Percentage of EGFR–AP-2 colocalization was calculated as the ratio of EGFR puncta with a nearest AP-2 neighbor within the specified distance to the total number of EGFR puncta (code available at: https://github.com/marybello/KNN_colocalization). Statistical analyses and visualizations were performed using GraphPad Prism (GraphPad Software).

### Western blotting

Proteins were extracted using RIPA buffer supplemented with protease and phosphatase inhibitors (Thermo Fisher Scientific). Protein concentrations were determined using the BCA protein assay (Pierce, Thermo Fisher Scientific). Samples were resolved by SDS-PAGE on 4 to 15% polyacrylamide gels under reducing conditions at 150 V for 40 min and then electrophoretically transferred onto 0.45-μm PVDF membranes (Millipore) using a wet transfer system. The blots were washed three times in Tris-buffered saline containing 0.1% Tween 20 (TBST), blocked with 3% BSA in TBST for 1 h, and incubated with primary antibodies overnight at 4 °C. The primary antibodies used were as follows: rabbit anti-EGFR (1:1000; #4267, Cell Signaling Technology), rabbit anti-phospho-EGFR Y1068 (1:1000; #2236, Cell Signaling Technology), and mouse anti-β-actin (1:100; sc-47778, Santa Cruz Biotechnology). Following primary incubation and washing, membranes were probed with HRP-conjugated secondary antibodies (1:3000; #7074, Cell Signaling Technology) for 1 h at room temperature. Protein bands were visualized using enhanced chemiluminescence (ECL) reagents (Thermo Fisher Scientific) and detected with an iBright 1500 imaging system (Thermo Fisher Scientific). Band intensities were quantified by densitometry using ImageJ software (National Institutes of Health).

## Data availability

All data supporting the findings of this study are contained within the manuscript and its supporting information. Raw data are available from the corresponding author upon reasonable request.

## Supporting information

This article contains supporting information.

## Conflict of interest

The authors declare that they have no conflicts of interest with the contents of this article.
