# Supplementary material for: Oncogenic EGFR mutants differentially alter dimerization, adaptor protein engagement, and clathrin-mediated internalization
Source: J Biol Chem. 2026 Jul 21;302(9):113353. doi: 10.1016/j.jbc.2026.113353 (PMC13499156; doi:10.1016/j.jbc.2026.113353)
Supplement: Supplementary file 1 [file mmc1.docx]

# **Supplementary Information for:**

Oncogenic EGFR Mutants Differentially Alter Dimerization, Adaptor Protein Engagement, and Clathrin-Mediated Internalization

*Maryam Bello-Hassan, Pradeep Kumar Singh, Adam W. Smith*

Department of Chemistry & Biochemistry, Texas Tech University, Lubbock, TX 79409, USA

**Figure S1. EGFR density does not account for mutation-specific Fc differences.** Scatter plots show the relationship between density and fraction correlated data for wild-type (WT) and EGFR mutant variants (L858R, E746-A750del, and L747-A750>P). Each dot represents an individual cell. Spearman correlation analysis revealed no significant correlation between EGFR density and the fraction of correlation for EGFR^WT^ (r = -0.1032, p = 0.3445), EGFR^E746-A750del^ (r = -0.05242, p = 0.5814), and EGFR^L747-A750>P^ (r = -0.1487, p = 0.1418). A statistically significant negative correlation was observed for EGFR^L858R^ (r = -0.4076, p = 0.002).

**Figure S2: Baseline surface fluorescence intensity of EGFR constructs prior to ligand stimulation.**

Mean baseline fluorescence intensities of EGFR–eGFP constructs (EGFRWT, L858R, E746–A750del, and L747–A750>P) in SUM159 cells were quantified from TIRF images prior to EGF addition. Raw fluorescence values were measured in ImageJ from multiple membrane regions of interest (ROIs) per cell following background subtraction. Data represent mean ± SEM. Statistical comparisons were performed using one-way ANOVA in GraphPad Prism and revealed no significant differences in baseline fluorescence intensity among constructs (ns), indicating comparable expression levels across EGFR variants prior to ligand stimulation.

**Figure S3. Baseline surface EGFR levels do not account for mutation-specific internalization differences.** Scatter plots show the relationship between baseline background-corrected fluorescence intensity (A.U.) and surface EGFR intensity remaining at 30 minutes (%) for wild-type (WT) and EGFR mutant variants (L858R, E746-A750del, and L747-A750>P). Each dot represents an individual cell. Spearman correlation analysis revealed no significant correlation between initial EGFR expression levels and the extent of receptor downregulation for EGFR^WT^ (r = 0.1048, p = 0.5144), EGFR^E746-A750del^ (r = 0.04964, p = 0.7432), and EGFR^L747-A750>P^ (r = 0.1103, p = 0.4981). A weak but statistically significant positive correlation was observed for EGFR^L858R^ (r = 0.3695, p = 0.0136).

**Figure S4: Time lapse images of unstimulated EGFR^WT^-eGFP in SUM159 cells.**

Representative TIRF microscopy time series showing the distribution of EGFR WT–eGFP at the plasma membrane under basal (unstimulated) conditions. Cells expressing EGFR WT–eGFP were imaged over time, and images correspond to the indicated time points (0–30 min). Scale bar = 10 µm.

**Figure S5: EGFR^WT^-AP2 colocalization before and after EGF stimulation:** Representative TIRF microscopy time series showing the dynamics of EGFR^WT^ and AP-2 at the plasma membrane following ligand stimulation. Cells expressing EGFR^WT^ (cyan) and AP-2 (red) were imaged over time before and after addition of EGF. Images correspond to the indicated time points (0–30 min) following stimulation. The top row shows EGFR^WT^ signal, the middle row shows AP-2, and the bottom row shows the merged channels highlighting sites of receptor–adaptor colocalization. Scale bar = 10 µm.

**Figure S6: EGFR^L858R^-AP2 colocalization before and after EGF stimulation.** Representative TIRF microscopy time series showing the dynamics of EGFR^L858R^ and AP-2 at the plasma membrane following ligand stimulation. Cells expressing EGFR^L858R^ (cyan) and AP-2 (red) were imaged over time before and after addition of EGF. Images correspond to the indicated time points (0–30 min). The top row shows EGFR^L858R^ signal, the middle row shows AP-2, and the bottom row shows the merged channels highlighting sites of receptor–adaptor colocalization. Scale bar = 10 µm.

**Figure S7: EGFR^L747-A750>P^-AP2 colocalization before and after EGF stimulation.**

Representative TIRF microscopy time series showing the dynamics of EGFR^L747–A750>P^ and AP-2 at the plasma membrane following ligand stimulation. Cells expressing EGFR^L747–A750>P^ (cyan) and AP-2 (red) were imaged over time before and after addition of EGF. Images correspond to the indicated time points (0–30 min) following stimulation. The top row shows EGFR^L747–A750>P^ signal, the middle row shows AP-2, and the bottom row shows the merged channels highlighting sites of receptor–adaptor colocalization. Scale bar = 10 µm.

**Figure S8: EGFR^E746-A750del^-AP2 colocalization before and after EGF stimulation.** Representative TIRF microscopy time series showing the dynamics of EGFR^E746–A750del^ and AP-2 at the plasma membrane following ligand stimulation. Cells expressing EGFR^E746–A750del^ (cyan) and AP-2 (red) were imaged over time before and after addition of EGF. Images correspond to the indicated time points (0–30 min) following stimulation. The top row shows EGFR^E746–A750del^ signal, the middle row shows AP-2, and the bottom row shows the merged channels highlighting sites of receptor–adaptor colocalization. Scale bar = 10 µm.

**Figure S9: Baseline puncta count for EGFR WT vs mutants.** Quantification of EGFR puncta at the plasma membrane under basal (unstimulated) conditions for EGFR WT and oncogenic mutants. Puncta were detected from TIRF microscopy images using Trackmate (Fiji) and counted per cell. Bars represent the mean puncta count per cell ± SEM. Statistical significance was determined using one-way ANOVA with multiple comparisons (*P* < 0.05, ***P* < 0.001).

**Figure S10: Basal and EGF-stimulated phosphorylation of EGFR wild type and oncogenic mutants.**

**(A)** COS7 cells stably expressing EGFR WT, L747–A750>P, or L858R, or left untransfected, were analyzed under basal conditions by immunoblotting for total EGFR, phospho-EGFR (pEGFR Y1068), and β-actin (loading control). **(B)** Quantification of tEGFR normalized to β-actin from COS7 cells. Data represent mean ± SEM from three independent experiments. **(C)** Quantification of pEGFR normalized to total EGFR from COS7 cells. Data represent mean ± SEM from three independent experiments. **(D)** SUM159 cells stably expressing EGFR WT or the indicated mutants (L858R, E746–A750del, L747–A750>P), or left untransfected, were either untreated (−) or stimulated with EGF for 5 minutes. (+). Whole-cell lysates were analyzed by immunoblotting for total EGFR, phospho-EGFR (pEGFR Y1068), and actin (loading control). **(E)** Quantification of tEGFR normalized to β-actin from SUM159 cells. Data represent mean ± SEM from three independent experiments. **(F)** Quantification of pEGFR normalized to total EGFR from SUM159 cells. Data represent mean ± SEM from three independent experiments.

**Figure S11: Chromatic alignment validation using multicolor TetraSpeck microspheres.** Multicolor fluorescent TetraSpeck microspheres were imaged under the same TIRF microscopy conditions used for cellular experiments to assess channel alignment and spatial registration between imaging channels. Because each bead emits fluorescence across multiple wavelengths, correctly aligned optical paths produce coincident signals appearing as overlapping red and green spots. Representative fields are shown demonstrating minimal spatial offset between channels. These images were used to confirm accurate channel registration prior to quantitative colocalization analysis. Scale bars: 5 µm.
